# Correction: Mechanistic insights into Ag^+^ induced size-growth from [Au_6_(DPPP)_4_]^2+^ to [Au_7_(DPPP)_4_]^2+^ clusters

**DOI:** 10.1039/d4na90024c

**Published:** 2024-02-21

**Authors:** Ying Lv, Xiaohang Wu, Shuping He, Haizhu Yu

**Affiliations:** a Department of Chemistry, Centre for Atomic Engineering of Advanced Materials, Anhui Province Key Laboratory of Chemistry for Inorganic/Organic Hybrid Functionalized Materials, Key Laboratory of Structure and Functional Regulation of Hybrid Materials of Ministry of Education, Anhui University Hefei 230601 Anhui P. R. China yuhaizhu@ahu.edu.cn; b Institute of Energy, Hefei Comprehensive National Science Center Hefei Anhui 230031 P. R. China

## Abstract

Correction for ‘Mechanistic insights into Ag^+^ induced size-growth from [Au_6_(DPPP)_4_]^2+^ to [Au_7_(DPPP)_4_]^2+^ clusters’ by Ying Lv *et al.*, *Nanoscale Adv.*, 2022, **4**, 3737–3744, https://doi.org/10.1039/D2NA00301E.

The authors regret that Fig. 2 and Fig. 3 in the published paper are identical. The correct Fig. 2 is given here.

**Fig. 2:**
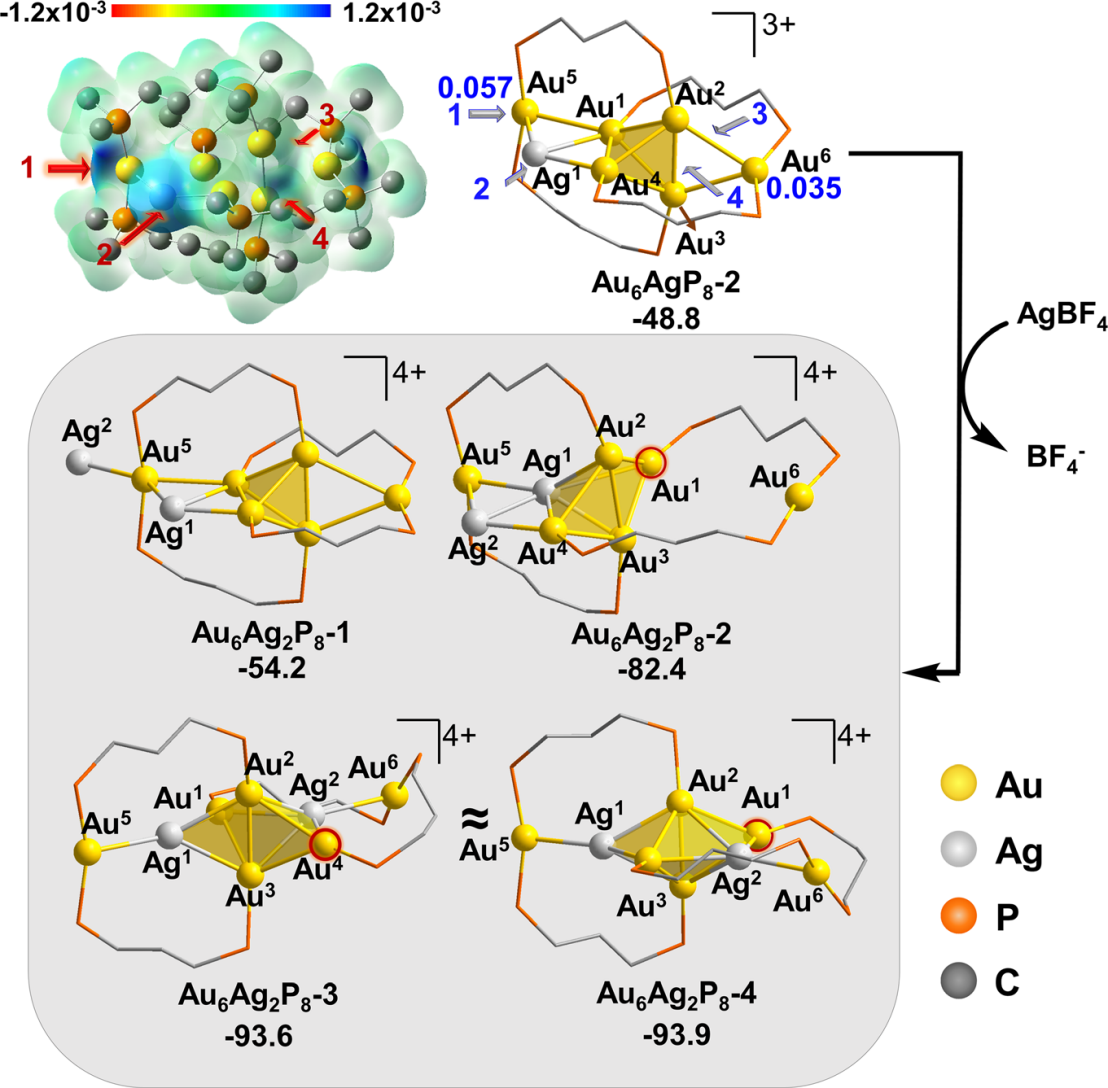
The isosurface of the *f*^−^ for **Au_6_AgP_8_-2**, using the width of Gaussian function of 0.01 au and the energy (in kcal mol^−1^) and structural changes for the doping of second Ag^+^ into **Au_6_AgP_8_-2**. The Hirshfeld charge of Au^5/6^ in starting structure is given in blue and bold.

The Royal Society of Chemistry apologises for these errors and any consequent inconvenience to authors and readers.

## Supplementary Material

